# Performance of malaria microscopy external quality assessment and networking among health facilities in west Amhara region, Ethiopia

**DOI:** 10.1186/s12879-020-05077-5

**Published:** 2020-05-19

**Authors:** Banchamlak Tegegne, Kefale Ejigu, Getaneh Alemu, Yeshimebet Fetene, Kindye Endaylalu, Mulatu Melese

**Affiliations:** 1 Amhara Public Health Institute, P.O. BOX: 477, Bahir Dar, Ethiopia; 2 Department of Medical Laboratory Science, Bahir Dar University, Bahir Dar, Ethiopia

**Keywords:** External quality assurance, Re-checking, Test agreement

## Abstract

**Background:**

Microscopic examination of peripheral blood smear produces reliable results both about the malaria infection status and level of parasitemia. However, test results are affected by skill of the laboratory personnel, workload, condition of microscopes and quality of laboratory supplies. Therefore, continuous monitoring of the performance of laboratories is of pivotal importance in order to make timely correction.

**Methods:**

A facility based cross-sectional study was conducted from July 2017 to July 2019 to assess malaria microscopy performance among thirty malaria diagnostic laboratories in west Amhara region. Thirty slides were collected from participating laboratories every quarter. Collected slides were taken to Amhara Public Health Institute reference laboratory and re-checked by malaria microscopists who were blind to the results from health facilities. Percentage of test agreement, rates of false positive, false negative and species misdiagnosis were calculated using Excel 2010.

**Results:**

Among a total of 6689 slides re-checked, results of 6146 slides were the same with that of participating laboratories. The test agreement was 97.31 and 94.6% for parasite detection and species identification, respectively. Variations in the overall performance of individual laboratories were seen within a range of 81.55 to 97.27% test agreement. Results of 543 (8.12%) slides were discordant, of which 363 (5.4%), 93 (1.4%) and 87 (1.3%) slides were due to species misdiagnosis, false positive and false negative results, respectively.

**Conclusion:**

There was good test agreement between participated laboratories and Amhara Public Health Institute. More accurate performance is expected as the country is tracking to malaria elimination. Hence, further strengthening the external quality assurance program is recommended.

## Background

Malaria is a febrile disease caused by intracellular haemoparasites of the genus *Plasmodium* [1]. Despite tremendous efforts have been made to combat malaria, the disease still remains a global public health problem. In 2018, about 228 million cases and 405 thousand deaths were reported globally. About 67% of the deaths occurred among under-5 children [1]. The burden outweighs in the tropics and subtropics that the World Health Organization (WHO) African region contributes for 93% of the cases and 94% of the deaths [1]. Based on the current stratification in Ethiopia, 60% of the population lives in risk areas; altitude and rain fall being important indicators [2]. About 1.5 million confirmed cases and 356 deaths were reported in 2017 in the country. Besides, *P. falciparum* (the most virulent species) is more prevalent in the country infecting 69% of confirmed cases in the same year [3]. Moreover, *Anopheles arabiansis,* a species responsible for malaria epidemics, is the primary vector transmitting malaria in Ethiopia, which makes the country prone to outbreaks [1, 4].

Early diagnosis and treatment of cases helps to avoid complication and death due to malaria. It also decreases both parasite transmission and misuse of anti-malaria drugs [3, 4]. Definitive diagnosis based on clinical manifestations is not possible; because many of the signs and symptoms overlap with that of other febrile illnesses [5, 6]. Accordingly, the Ethiopian malaria national strategic plan states that 100% of suspected cases should be diagnosed in the laboratory within 24 h of fever onset [2].

Laboratory diagnosis of malaria is made by rapid diagnostic tests, blood film microscopy or molecular techniques [7]. In health facilities equipped with clinical laboratory, microscopic examination of stained thin and thick peripheral blood smears is the most commonly practiced technique; because the technique is easily accessible and affordable in peripheral laboratories. It also produces reliable results about both the infection status and parasitemia level [8]. Microscopy has a sensitivity of detecting as few as 5–10 parasites/μl of blood [9]. However, the test result is affected by multiple factors including skill of the laboratory workforce, workload, condition of microscopes and quality of laboratory supplies [9]. Hence, it is of primary concern to ensure diagnostic services: which provide accurate results; are administered by competent and motivated staff supported by effective training, supervision and quality control. Diagnostic laboratories should also be supported by a logistic system to provide and maintain adequate supplies of reagents and equipments [6].

Assessment of the diagnostic performance could be made by involving laboratories in External Quality Assurance (EQA) program [9]. It is a vital tool for identifying gaps in laboratory performance and targeting areas for improvement. It can be performed through panel testing, blind re-checking and/or onsite evaluation [10]. In Ethiopia, the regional central laboratories are mandated to perform EQA in health facilities of respective regions. Hence, Amhara Public Health Institute (APHI) engages health facilities located in the region. Compiled data on EQA performance of involving laboratories helps to inform common problems and recommend for corrective actions. It also shows the impact of EQA on malaria microscopy performance of health laboratories. A similar study conducted in west Amhara before 5 years revealed a mean test agreement of 96.6% with 2.63, 0.7 and 3.4% of re-checked slides reporting false positive, false negative and species mis-diagnosis results, respectively [11]; however the results might not be consistent as there is difference in laboratory staff (due to turnover and recruitment), training, patient flow, quality of supplies and test procedures. Therefore, the aim of the present study was to show the recent 2 years malaria microscopy performance of public health facility laboratories in west Amhara region as assessed through blind rechecking.

## Methods

### Study design and area

A facility based cross-sectional study was conducted from July 2017 to July 2019 among thirty malaria diagnostic health facilities in west Amhara region. There are 39 hospitals, 523 health centers and one public health institute (APHI) in the region. Amhara Public Health Institute directly conducts EQA for malaria diagnosis in selected hospital and health center laboratories in the region. Selection of laboratories is based on availability and scope of human resource, strength of internal quality control, commitment of the health facility and laboratory management to perform EQA, training on laboratory quality management and safety, geographical proximity for supporting peripheral laboratories and proficiency of the laboratory from previous EQA participation. Participating laboratories, in turn, serve as ‘EQA centers’ for peripheral health laboratories under their cluster. Hence, all health laboratories in Amhara region are networked to APHI malaria EQA program. Head quarter of the institute is located at Bahir Dar city and has one branch at Dessie. Both the head quarter and Dessie branch perform similar EQA activities. The Bahir Dar site performs EQA among health facilities in the western Amhara region and the Dessie site recruits health facilities from eastern region. In the present study, thirty facilities in the western region were recruited. These thirty health facilities were addressed by blind rechecking program quarterly (every 3 months).

### Study procedure

Thirty health facility laboratories (24 hospitals and 6 health centers) have been participating in the EQA program for malaria diagnosis performed by Bahir Dar site of APHI since 2016; all were included in the present study. The malaria diagnosis performance was assessed through blind re-checking of stained blood film slides collected from laboratories and onsite supervision. Participating laboratories were requested to store both positive and negative slides. Then, malaria laboratory experts from APHI went to the sites at every quarter year and collected 30 slides following the WHO recommendation [9]. Selection of slides was made from the registration log book by systematic random sampling as per the national malaria diagnosis external quality assessment scheme guideline [12]. Collected slides were taken to APHI reference laboratory and re-checked by malaria microscopists who were blind to results from health facilities. Discrepant results were re-examined again by a senior quality officer and his/her finding was taken as the final result. After each round, malaria laboratory experts from APHI visited participating laboratories to show errors of discordant slides and to give practical training so that the performance of laboratories will be improved. The institute has also sent a written feedback to all participating laboratories. The feedback contains information about discordant slides, smearing and staining quality of slides and gaps to be improved. Discordant management form was also administered to laboratory professionals where discordant results were reported. The form contains list of possible reasons for inaccurate microscopy results. In this way, EQA data collected for 8 consecutive quarters from July 2017 to July 2019 were included for the present study. Data were entered and analyzed in Microsoft Excel 2010. Percentages of agreement as well as rates of false positive, false negative and species misdiagnosis were calculated.

## Results

A total of 25 health facility laboratories (19 hospitals and 6 health centers) participated in all the 8 rounds of EQA. Three hospital laboratories participated in 7 rounds and two hospital laboratories participated in 6 and 5 rounds each. We have included data from all 30 health facilities collected between July 2017 and July 2019. A total of 6689 slides were collected and re-examined by malaria laboratory experts in APHI. Results of 6146 slides were the same with that of participating laboratories, to give an overall result agreement of 91.88%. Test agreements in parasite detection and species identification were 97.31 and 94.6%, respectively. Results of 543 (8.12%) slides were found to be discordant. Variations in the performance of individual laboratories were seen within a range of 81.55 to 97.27% result agreement (Table 1). Analysis of performance by EQA round revealed the lowest (88.73%) and the highest (96.30%) test agreements in rounds 3 and 5, respectively. Performance of laboratories didn’t show uniform trend in every round of EQA (Table 2). More than half of the discordant results (363 out of 543) were due to species misdiagnosis while 93 and 87 slides were false positive and false negative results, respectively. The highest frequency of species mis-diagnosis was seen in slides positive for mixed infection; 91 slides with mixed infection were reported as *P. vivax* (Table 3).

**Table 1:** Agreement of each participated laboratory and APHI slide readers in eight consecutive rounds of EQA from July 2017 to July 2019, west Amhara, Ethiopia

Lab	R1	R2	R3	R4	R5	R6	R7	R8	Total	Total Agreed	Total Discordant	% Agreement
1	30	30	30	30	30	30	30	30	240	225	15	94.2
2	30	30	30	30	30	30	30	30	240	220	20	91.67
3	30	30	30	30	30	30	30	30	240	220	20	91.67
4	29	29	30	30	30	30	30	30	238	221	17	92.86
5	30	30	30	30	30	29	30	30	239	228	11	95.4
6	30	30	30	30	30	30	30	30	240	220	20	91.67
7	30	30	30	30	30	17	30	30	227	216	11	95.15
8	11	26	30	30	30	30	30	16	203	197	6	97.04
9	30	30	30	30	30	29	30	30	239	220	19	92.05
10	30	30	30	30	30	30	30	30	240	220	20	91.67
11	30	30	30	30	30	28	30	30	238	220	18	92.44
12	24	20	30	0	30	30	0	30	164	147	17	89.63
13	24	30	30	30	30	30	30	0	204	185	19	90.69
14	19	25	30	0	30	30	30	30	194	170	24	87.63
15	30	30	30	30	30	30	30	20	230	220	10	95.66
16	30	30	30	30	30	30	30	30	240	220	20	91.67
17	30	30	30	30	30	27	30	30	237	218	19	91.98
18	30	30	30	30	30	30	30	30	240	220	20	91.67
19	30	30	30	30	30	30	30	30	240	225	15	93.75
20	30	30	27	30	15	30	19	30	211	191	20	90.52
21	30	30	30	30	30	30	30	30	240	221	19	92.08
22	30	30	30	30	30	26	14	30	220	214	6	97.27
23	24	30	30	30	16	30	9	30	199	173	26	86.93
24	27	29	30	30	30	23	29	30	228	207	21	90.79
25	27	30	30	30	30	30	29	30	236	214	22	90.68
26	30	30	30	30	30	24	30	30	234	226	8	96.58
27	25	30	30	30	30	14	24	30	213	200	13	93.9
28	20	30	29	30	30	20	30	30	219	189	30	86.3
29	26	30	30	30	0	30	30	30	206	168	38	81.55
30	30	30	30	30	0	0	30	0	150	131	19	87.33
Total	826	879	896	840	811	807	814	816	6689	6146	543	91.88

*R* Round

**Table 2:** Discordant results between participated laboratories and APHI malaria slide readers in each round of EQA from July 2017 to July 2019, west Amhara, Ethiopia

Round	Number of collected slides	Agreed Result N (%)	Total Discordant N (%)	False positive N (%)	False negative N (%)	Species mis-diagnosis N (%)
R1	826	757 (91.65)	69 (8.4)	17 (2.1)	11 (1.3)	41 (5.0)
R2	879	783 (89.08)	96 (10.9)	9 (1.0)	8 (0.9)	79 (9.0)
R3	896	795 (88.73)	101 (11.3)	17 (1.9)	15 (1.7)	69 (7.7)
R4	840	796 (94.76)	44 (5.2)	6 (0.7)	7 (0.8)	31 (3.7)
R5	811	781 (96.30)	30 (3.7)	9 (1.1)	6 (0.7)	15 (1.8)
R6	807	723 (89.59)	84 (10.4)	9 (1.1)	14 (1.7)	61 (7.6)
R7	814	761 (93.49)	53 (6.5)	13 (1.6)	13 (1.6)	27 (3.3)
R8	816	750 (91.91)	66 (8.1)	13 (1.6)	13 (11.6)	40 (4.9)
Total	6689	6146 (91.88)	543 (8.12)	93 (1.4)	87 (1.3)	363 (5.4)

**Table 3:** Species mis-diagnosis results between participated laboratories and APHI malaria slide readers in each round of EQA from July 2017 to July 2019, west Amhara, Ethiopia

Slide Result	R1	R2	R3	R4	R5	R6	R7	R8	Total
Pf as Pv	13	21	12	6	4	14	6	8	84
Pf as mixed	8	8	8	1	4	6	2	3	40
Pv as Pf	8	4	0	4	2	25	12	18	73
Pv as mixed	4	0	2	0	0	6	2	7	21
mixed as Pf	5	15	9	12	2	5	3	3	54
mixed as Pv	3	31	38	8	3	5	2	1	91
Pf as negative	6	4	7	2	4	5	4	5	37
Pv as negative	3	2	5	5	1	8	8	8	40
mixed as negative	2	2	3	0	1	1	1	0	10
negative as Pf	8	2	8	5	5	5	6	8	47
negative as Pv	9	6	9	1	3	4	7	4	43
negative as mixed	0	1	0	0	1	0	0	1	3
**Total**	69	96	101	44	30	84	53	66	543

*Pf Plasmodium falciparum*, *Pv Plasmodium vivax*

## Discussion

Early diagnosis of malaria plays an important role both for prompt treatment and transmission intervention. Ethiopia has set a malaria control strategic plan to be implemented from 2017 to 2020. Goals of the plan include reducing malaria cases by 40% (the baseline being 2016 data), maintain near zero deaths and implement malaria elimination in 239 districts by 2020. ‘Laboratory diagnosis of all cases within 24 hours of fever onset in 2017 and beyond’ was one of the strategic objectives to achieve the goals [4]. For accurate case detection and successful malaria elimination, quality of diagnosis is indispensable. Despite blood film microscopy is the gold standard technique, it is prone to errors in the smear preparation, staining, parasite detection, species identification and quantification phases. Therefore, periodic in service trainings are given to laboratory personnel and their performance is monitored through EQA programs by experts from central laboratories.

The ultimate goal of the EQA program is to enhance the quality of malaria diagnosis by improving the competency of laboratory personnel and quality of laboratory utilities [13]. Therefore, all health facility laboratories should be benefitted by participating in the program. However, implementing EQA directly managed at national or regional centers is too costy in terms of time, logistics and human power. This brings difficulty in sustained implementation of the program, especially in resource limited countries like Ethiopia. Considering this, APHI has decentralized the malaria EQA program since 2012. We believe other regions or countries will be benefitted if they adopt the decentralized and networked EQA implementation approach.

Despite it was planned that 30 slides were to be collected from each laboratory, less number or no slides were collected in some rounds (Table 1). This was due to political instability in areas where respective health laboratories are located. The mean test agreement in detecting malaria parasites in the present study (97.31%) was slightly higher than previous results from Amhara region of Ethiopia (96.6%) [11], and slightly lower than Pakistan (99.0–99.5%) [14]. On the other side the test agreement was significantly higher than recent results of 78, 88 and 91.7% from Oromia region of Ethiopia [15], Hawassa [16] and Addis Ababa [17], respectively. Variations in laboratory workload, training and assessment methods might bring the difference. Periodic in service training given to laboratory personnel accompanied with close supervision and feedback after each round of EQA is thought to bring the high accuracy in detecting malaria parasites in the region.

Rate of false positive results (1.4%) was lower than previous findings of 2, 2.64 and 4.05%, 7.8, 24.6 and 24.4% from Canada [18], west Amhara, Ethiopia [11], Addis Ababa, Ethiopia [17], USA [19], Congo [20] and Oromia, Ethiopia [15], respectively. Similarly, frequency of false negative reporting was also low (1.3%) in the present study, implying that the overall performance of health facilities in malaria parasite detection is acceptably good. However, as the country is moving from malaria control to elimination, any non-zero report of false positive and/or false negative will be significant [4]. Data from discordant management form shows that high workload contributes for false negative results. For example 25% of laboratory professionals reporting false negative results responded that they frequently observe less than 100 fields before reporting negative slides due to high workload. The national malaria guideline recommends a minimum of 100 fields should be observed in the thick smear before reporting negative results [21]. Other factors contributing for false negative results were lack of training, failure to follow standard operating procedures, poor quality supplies and clerical errors. Similarly, experience and training gaps and clerical errors were the two common causes for false positive reports.

Treatment of malaria varies according to the infecting *Plasmodium* species [21]. Therefore, laboratories should identify and report species correctly. The proportion of species mis-diagnosis in the present study (5.4%) goes in line with previous results of 3.4% in the same study area [11] and it is much lower than previous studies from Hawassa [16] and Oromia [15] where the laboratory professionals correctly identified the species in 74.3 and 44.6% of malaria positive slides during panel testing, respectively. The discrepancy might be due to difference in the method of assessment and the status of EQA and other supportive activities from reference laboratories. In general, different factors encountering at the pre-analytical, analytical and post-analytical steps of malaria microscopy equally contribute for discordant results. In the present study, correct reporting of *P. falciparum, P. vivax* and mixed infection was a major problem identified. Failure to prepare and examine thin film might be a possible reason as 25% of laboratory professionals reporting species mis-diagnosis responded that they identify species from thick blood film. Similarly 20 and 10% of professionals reported gap in training and experience, respectively. Quality of smearing and staining also contribute for correct parasite detection and species identification, which were not assessed due to inconsistency of such data.

As compared to a similar study conducted in west Amhara before 5 years, the overall result agreement of laboratories shows a slight improvement (97.3% vs 96.6%). Similarly, rate of false positive results decreases from 2.64 to 1.4% [11]. Continuous in-service trainings given by APHI to laboratory professionals and consistent participation in EQA program are attributed for such improvement. On the contrary, rate of false negative (0.7% vs 1.3%) and species mis-diagnosis (3.4% vs 5.4%) slightly increased in the present study as compared to the previous results [11]. Turnover of trained and experienced staff and increased workload are believed to be reasons for the increment of such errors.

## Conclusion

There was good agreement in parasite detection (97.31%) and species identification (94.6%) between peripheral laboratories and malaria experts in APHI in west Amhara region. However, there was no regular trend of improvement in overall performance across the eight rounds of EQA. More accurate malaria microscopy performance is also expected as the country is tracking to malaria elimination. Hence, ensuring optimum workload and strengthening the EQA program by integrating re-checking with onsite evaluation is recommended.

## Data Availability

The original data for this study is available from the corresponding author.

## Abbreviations

APHI: Amhara Public Health Institute
EQA: External Quality Assurance
WHO: World Health Organization
